# Collagen crosslinking in the management of microbial keratitis

**Published:** 2016

**Authors:** Barac Ileana Ramona, Corbu Catalina, Merticariu Andrei, Stefan Daciana, Tataru Calin

**Affiliations:** * Emergency Eye Hospital Bucharest, Romania; ** ”Carol Davila” University of Medicine and Pharmacy, Bucharest, Romania

**Keywords:** crosslinking, corneal ulcer, microbial keratitis

## Abstract

Objective – the evaluation of the efficiency of corneal cross linking in the management of corneal ulcers.

Method - a prospective study that included 10 patients, 10 eyes, with chronic corneal ulcer, bacterial and/ or fungal. The patients were divided into two groups. Group A included 5 patients with unperforated corneal ulcer and group B included 5 patients with perforated corneal ulcer. These patients were treated with general and local antibiotic and antifungal drugs, but the response was poor after two weeks. The crosslinking procedure was performed and the local treatment was continued for two weeks. An additional partial or total conjunctival coverage was done on group B. Patients were evaluated after 3 days, one week, two weeks, one month, 3 months, 6 months and one year after the procedure. Slit lamp and tomographic aspects of the cornea were assessed as well as the visual acuity. Results - all ten patients experienced a decrease in pain from the first postoperative day. The ulcer healed by more than 50% in the first week in 3 patients from group A and closed completely after one month for 4 patients in group A, respectively 4 patients in group B. Hypopyon did not reappear after the crosslinking procedure in group A. However, it did persist in one patient from group B. Postoperative results were the same at 3, 6 and 12 months after the procedure. An opacification of the lens was observed in 3 patients after crosslinking. There were not any intra operative and postoperative complications.

Conclusion - Corneal crosslinking is efficient in the management of patients with chronic corneal ulcer.

## Introduction

Infected corneal ulceration is an affliction frequently met in the ophthalmic pathology and has a chronic (trenant) evolution with a negative outcome in many cases, despite the treatment. Corneal perforation, hypopyon, and endophthalmitis are negative markers in the outcome of the ulcers, which could even go as far as the eyeball evisceration. Long antimicrobial treatment can lead to microbial mutation with development of resistant strains [**1**].

Corneal crosslinking is usually performed for the treatment of keratoconus, but many studies have demonstrated its efficiency in patients with chronic corneal ulcer [**2**-**5**]. Corneal crosslinking with topic riboflavin drops and exposure to UVA does a photopolymerisation of the stromal collagen fibers with the increase of corneal rigidity and resistance for ectasia. Through this kind of procedure, the biomechanical resistance of the cornea also increases by increasing the collagen fiber diameter [**6**].

Riboflavin crosslinking increases the resistance of corneal fibers to the bacterial collagenase, trypsin and pepsin, this biochemical effect adding to the biomechanical effect of the procedure in the treatment of infected ulcers [**7**,**8**].

The bactericidal effect of UVA is well known, the free radicals produced by crosslinking interfering with the integrity of the microbial cell walls [**9**].

## Materials and method

This study included 10 patients, 10 eyes with chronic corneal ulcer, microbial and/ or fungal in nature, who were hospitalized in the Emergency Eye Hospital and Clinic in Bucharest, between 01.01.2014 and 10.10.2015. Patients were divided into two groups. Group A included 5 patients with unperforated chronic corneal ulcer and group B included 5 patients with perforated ulcer. These patients had local, general antibiotic and antifungal treatment, but without any improvement after two weeks (inclusion criteria). The crosslinking procedure was performed after at least two weeks of treatment and continued with the local drops for another two weeks. A total conjunctival coverage was also performed for patients in group B. The patients were evaluated after 3 days, one week, two weeks, two months, one month, three months, six months, and one year post-procedure. The slit lamp and tomographic aspect of the cornea, as well as the visual acuity, were assessed.

A conjunctival discharge evaluation with bacterial culture and sensitivity test, as well as a fungal culture, was done for all the patients. The general treatment consisted of a broad spectrum latest generation antibiotic and antifungal; the local treatment consisted of a fortified antibiotic and antifungal drops and also atropine. Patients with a poor response after a two weeks treatment were included in the study, a session of corneal crosslinking was done for them, and a conjunctival coverage was done for group B.

## The procedure

Corneal crosslinking was performed with topical anesthesia by using 0.5% proparacaine drops. Diluted betadine drops with an action time of 3 minutes were used for local disinfection. Before starting the procedure, a 0.1% riboflavin and dextran solution was used for corneal impregnation, with an exposure time of 30 minutes, one drop at every 3 minutes. The cornea was then exposed to UVA 365nm light and an intermediate program with 5,4J/cm2 was performed for 10 minutes. As the exposure went on, additional impregnation with riboflavin was done, one drop at every minute. The short span exposure was selected for preventing an increased corneal hypoxia.

Local retrobulbar anesthesia was used for patients in Group B. After the UVA exposure, the total conjunctival coverage was performed and daily eye closure was done for one week. Patients continued the local eye drop treatment for 2 weeks.

## Results

Group A consisted of 5 patients with chronic corneal ulcer with hypopyon. Their ages were between 21 and 55 and all of them were males; one patient had mild myopia and was a contact lens wearer, the other 4 patients from this group had no other ocular conditions, all of them with no pathological medical history.

There was a decrease in pain the first day after the procedure for all 5 patients from Group A. In 3 patients from group A, the ulcer submitted more than 50% in the first week and for 4 patients from this group, it closed completely after one month. There was no hypopyon remission after the procedure. Postoperative results were the same at 3, 6, and 12 months after. Visual acuity improved for 4 patients.

Group B consisted of 5 patients, one female and 4 males, with perforated corneal ulcer and hypopyon. Their ages were between 49 and 65; one patient had had a cataract surgery two years before, with a lens implant, and 4 of them were without any other ocular conditions; 2 patients were hypertensive, one had COPD.

For all the patients in Group B, pain decreased from the first day post procedure. Their general state improved significantly. The ulcer remitted within one month after the procedure for 4 of the patients. The hypopyon persisted for only one patient. Opacification of the lens was observed in 3 patients. There were no intra- and post- operative complications. Visual acuity improved for 4 out of 5 patients in this group.

## Conclusions

All 10 patients experienced a decrease in pain from the first day after the procedure, their general state improving as well.

The corneal ulcer closed completely after one month in 8 patients, 4 from group A and 4 from group B.

In conclusion, corneal crosslinking is an efficient solution in the treatment of patients with chronic corneal ulcer.

**Fig. 1:**
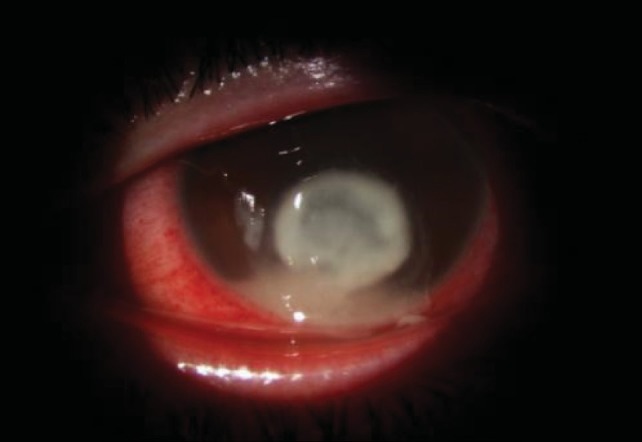
Corneal ulcer with hypopyon

**Fig. 2:**
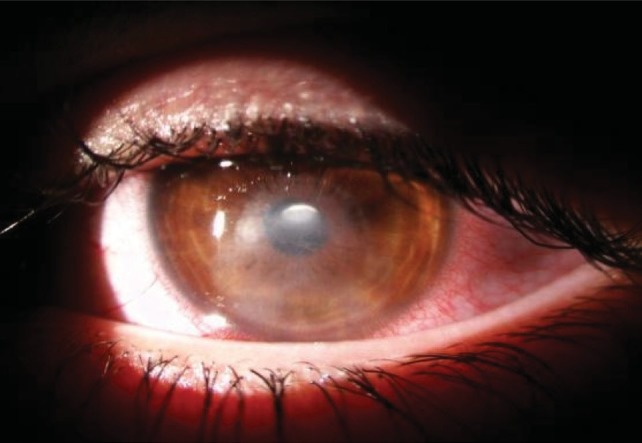
After 3 weeks

**Fig. 3:**
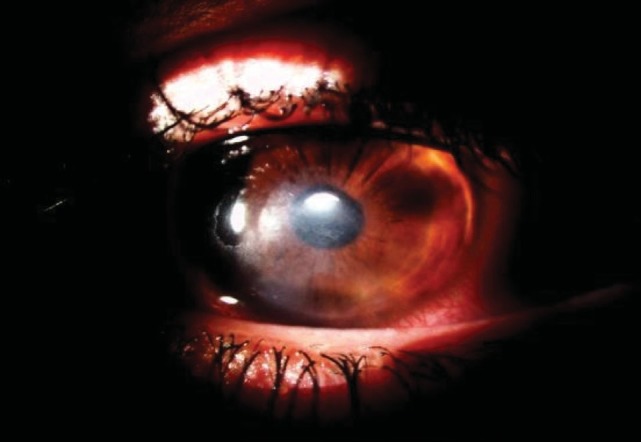
After 3 months
